# The promises and perils of the neuroscience of creativity

**DOI:** 10.3389/fnhum.2013.00246

**Published:** 2013-06-05

**Authors:** Anna Abraham

**Affiliations:** ^1^Department of Community Medicine and Behavioural Sciences, Faculty of Medicine, Kuwait UniversityJabriya, Kuwait; ^2^Department of Clinical Psychology, Justus Liebig University GiessenGiessen, Germany

**Keywords:** creative cognition, normative cognition, creative neurocognition, cognitive neuroscience, definitions, approaches, conceptual limitations, technical limitations

## Abstract

Our ability to think creatively is one of the factors that generates excitement in our lives as it introduces novelty and opens up new possibilities to our awareness which in turn lead to developments in a variety of fields from science and technology to art and culture. While research on the influence of biologically-based variables on creativity has a long history, the advent of modern techniques for investigating brain structure and function in the past two decades have resulted in an exponential increase in the number of neuroscientific studies that have explored creativity. The field of creative neurocognition is a rapidly growing area of research that can appear chaotic and inaccessible because of the heterogeneity associated with the creativity construct and the many approaches through which it can be examined. There are also significant methodological and conceptual problems that are specific to the neuroscientific study of creativity that pose considerable limitations on our capacity to make true advances in understanding the brain basis of creativity. This article explores three key issues that need to be addressed so that barriers in the way of relevant progress being made within the field can be avoided.

- Are creativity neuroimaging paradigms optimal enough?
- What makes creative cognition different from normative cognition?
- Do we need to distinguish between types of creativity?

The immense capacity of human beings to be creative can be gleaned from virtually all realms of our lives whenever we generate original ideas, develop novel solutions to problems, or express ourselves in a unique and individual manner. Despite the vital importance of this complex ability for the progress of our species across all fronts of human development, we still lack fundamental knowledge about how creative thinking occurs. What makes some people appear more creative than others? How much control do we exert over the expression of our creative abilities? Why are we sometimes unable to think with originality despite strenuous efforts, yet at other times we experience the flow of creativity almost effortlessly? Can we train our mental faculties to become more creative? How much of our creative potential is biologically predetermined? These are some of the many questions that beg further exploration.

Among the many approaches that have been applied to investigate creativity, the neurobiological approach has a surprisingly long history. The characterization of the brain's response when generating novel responses was achieved through the use of direct and indirect global measures, such as electroencephalography (EEG) and lateralization paradigms (for reviews, see Miran and Miran, 1984; Martindale, 1999). In fact, the insights from these studies beginning in the 1960s and 1970s, about the role of alpha wave brain activity during creative thinking, or left brain versus right brain contributions during the creative act, continue to influence EEG and cerebral asymmetry research in creativity to this day (Lindell, 2011; Fink and Benedek, 2012).

The mass availability of neuroimaging techniques began in the 1990s and resulted in an exponential surge of neuroimaging-based research to the extent that questions from virtually all domains in psychology have been (or are being) explored. The field of creativity is no exception to this trend (for a review, see Dietrich and Kanso, 2010).

While adapting most experimental paradigms in psychology for exploration using functional neuroimaging techniques is not unduly problematic, the same cannot be said for paradigms from the field of creativity. There are unique problems, both theoretical and methodological, that surface when trying to adapt most creativity tasks for use in neuroimaging environments. These problems, although seldom openly discussed or acknowledged, have significant implications for developing our understanding of how creative thinking develops (Dietrich, 2007a; Sawyer, 2011; Abraham, 2012). This treatise will explore some of the most pertinent issues that can deter progress from being made in the field. These can be formulated in the form of three questions.

- Are creativity neuroimaging paradigms optimal enough?
- What makes creative cognition different from normative cognition?
- Do we need to distinguish between types of creativity?

## Are creativity neuroimaging paradigms optimal enough?

The best way to consider this question would be contrast the general paradigm-based differences, both technical and conceptual, between commonly used creative tasks and non-creative tasks of higher-order cognition from other domains that involve engaging one's imagination, such as future thinking or hypothetical reasoning, or other aspects of higher-order cognition, such as declarative memory retrieval (e.g., Zysset et al., 2002; Abraham et al., 2008; Abraham and von Cramon, 2009).

There are several differences in methodological protocols (Table 1). Let us take the case of the number of trials per condition and trial duration. Functional run(s) in a typical fMRI experiment are usually limited to about 40 min as a maximum. Each trial in any fMRI paradigm does not only include the stimulus events(s) and/or responses, but also pauses of varying duration (typically a few seconds long). So the trial lengths in fMRI experiments are always longer than those of behavioral experiments. There is of course a wide range of possibilities when it comes to trial duration, which depends on research question and the type of experimental design being adopted (e.g., event-related versus block). However, in general, within experimental designs used to assess creativity, the duration of a single trial is much longer compared to other cognitive paradigms. This is because the stimulus events themselves are presented over longer periods (around 8–20 s), and these are often followed by a lengthy response phase. So this prolongation of trial duration usually necessarily means that there are far fewer trials in total per condition in fMRI paradigms that assess creativity compared to those that assess other complex aspects of cognition. This can have a considerable impact on the power and efficiency of the experimental design and, consequently, the confidence with which we can interpret the resulting findings.

**Table 1:** **A generalized comparison of common methodological and conceptual differences between creative and non-creative tasks**.

	**Non-creative imagination task**	**Creative imagination task**
Trials: number per condition	>20	<20
Trial duration	<10 s	>10 s
Trial classification	Hit, miss, false alarm, correct rejection	Predetermined (not based on response type)
Response type	Close-ended	Open-ended
Response options	Binary	Non-binary
Response modality	Button press	Vocal responses or Button press
Response log	Tracked in real-time (as it happens)	Recorded after the fact
Control task: match to experimental task	High	Low
Cognitive event: determining start point	Often	Rare
Cognitive event: prompt on cue	Yes	No
Certainty regarding process activation	High	Low
Certainty regarding tested process	High response accuracy = better cognitive performance	Trying to be creative ≠ Being creative

The efficiency-power tradeoff (Liu et al., 2001) is an important factor to consider when devising fMRI experimental paradigms. Here, power refers to the ability to detect any activation in the brain and efficiency refers to the accuracy at determining the shape of the hemodynamic response function to stimuli^1^. The power of an fMRI experimental design is exponentially related to number of trials per condition (until around 100–150 trials), whereas efficiency is acceptable at around 25 trials per condition (Huettel and McCarthy, 2001; Desmond and Glover, 2002). The problem is that the use of less than 20 trials per condition is not uncommon within fMRI experimental designs of creativity.

A further problem is the appropriateness of the control task to the creative task in question. Most neuroimaging studies on creativity compare brain activation elicited during creative thinking (e.g., generate a story using three semantically unrelated words) with that of a less cognitively demanding control tasks (e.g., generate a story using three semantically related words) or unspecific states such as rest. The latter option is particularly problematic to employ as a baseline given its utter lack of specificity (Stark and Squire, 2001). Indeed, one the earliest neuroimaging studies on episodic memory even referred to the resting brain as “… a resource not only for the creative process, but also for meditational states, religious experiences, and dreams” and that its activities reflect “substrates of the creative process” (Andreasen et al., 1995, p. 1577, 1583). Less cognitively demanding control tasks are also suboptimal to use as comparisons to creative tasks as it is not possible under such circumstances to tease apart the components of ensuing brain activations resulting from increased cognitive control with that of creative idea generation. Recent studies have attempted to optimally control for differing difficulty levels between the creative and non-creative control tasks through the use of different strategies (Abraham et al., 2012b; Aziz-Zadeh et al., 2012), but it is often at the expense of having a control task (s) that is qualitatively very different from the creative task.

Even at the level of response, creativity researchers face unique problems in implementation. Typically most creativity tasks are non-binary (cannot be reduced to yes/no decisions), open-ended (involve more than one response), subjective (there are no correct or incorrect responses as long as they are valid), and involve movement (spoken, written or drawn). Moreover, responses are rarely logged in “real-time,” i.e., as and when each creative response is generated. Typically, the participants are exposed to a stimulus cue, which indicates the task that they have to undertake over an extended period of time without moving (e.g., think of as many uses as you can for a “brick”). Following that, they are usually given a response cue after which they can indicate their responses within the scanner during temporally isolated response phases via either vocal (single word or more elaborate articulations) or button press responses. This manner of response isolation is for the purpose of minimizing movement-related problems (unspecific brain activations and potential motion artifacts) from affecting the pattern of findings. However, because such response isolation strategies are not foolproof, another alternative is to not have any actual responses during the imaging session but instead to instruct subjects in advance that they will be required to report their generated uses after the experiment. The problem of this approach is that one cannot be certain that the subjects are actually following task instructions during the imaging session, nor can one be sure that the responses are untainted by forgetting or post-experiment elaboration (for a compromise between both approaches and a lengthy discussion on this topic see, Abraham et al., 2012b).

Another problem with not having responses logged and coded in real time is that it is challenging to determine whether the trial can really be classified as creative or not. In a memory recall task, for instance, one can assess a subject's performance based on objective, close-ended, binary responses that can be instantly classified (following signal-detection theory, for instance) in the form of hits, misses, correct rejections and false alarms. The same cannot be done for creativity as the responses are assessed after the fact in terms of levels of creativity. What is more, each trial can be associated with multiple responses. In the absence of the on-line logging of responses, there is no way of knowing at which points during the extended trial the uses were generated, much less determine when the most creative of all the uses was produced.

The unfortunate truth is that one is particularly dependent on behavioral responses in creativity tasks because the stimulus event or task cue does not automatically evoke the necessary cognitive processes under study. Creativity cannot simply be prompted in a manner that is reliable or valid. This is one of the factors that set creative thinking apart most other aspects of cognition and renders it incredibly challenging to investigate. If asked whether you cut your hair last week or know the capital of Iceland, the question in itself would suffice as a prompt to evoke a response (yes/no). This is not so when you have to generate new uses for objects or create an original story or manipulate images to form a meaningful yet novel gestalt. So in the absence of a behavioral marker, we do not know the starting point of the cognitive event in question (generation of the creative idea) and analyses of the brain's response in relation to the actual point of creative insight cannot be conducted without this information.

Moreover, within an extended period ranging from a couple of seconds to almost half a minute when subjects have to generate novel uses for an object, the brain activation during that period reflects a mixture of brain regions that are engaged while developing strategies to solve the problem in conjunction with those involved in the actual generation of the solution. To make matters worse, the generated solution may be valid or invalid as well as creative or uncreative. And none of these factors can be effectively teased apart in such designs (e.g., strategy versus solution; creative versus uncreative solutions). Although several researchers specifically instruct their subjects to “be creative”, this is not a simple instruction to follow and is easier said than done. Merely instructing people to be creative does not guarantee their ability or success to do so. Notwithstanding the problem of definition of what it means to be creative (which will be explored later), our brains are not immune to the “path of least-resistance”, which is the overwhelming tendency, owing to our cost-effective brains, to follow the cognitively least demanding route in in generative situations (Ward, 1994).

These technical limitations of creativity paradigms are related to a much larger conceptual problem, namely that trying to be creative is *not* the same as being creative—a fact that all of us are likely to be keenly aware of from personal experience. While it is certainly useful to assess the pattern of brain response when people are trying to be creative in order to understand what happens in the brain when people are being creative, it is important that we, at the very least, remain cognizant of this crucial distinction and let it guide our interpretations of data accordingly.

So what does this admittedly daunting picture tell us about the state of affairs in the neuroimaging of creativity?

It illustrates that challenging times lie ahead and that the field is in need of a major paradigm-shift. While the extension of other cognitive paradigms to neuroscience may be unproblematic, the simple truth is that this cannot be done with the same degree of ease in the case of creativity.

This does not mean that creative thinking cannot be studied using neuroscientific techniques. But any technique, no matter how promising it may appear, is useless if the experimental designs are suboptimal. We need to devise experimental designs that are optimized for use in neuroscientific setups to investigate different facets of creative thinking. Or indeed, in the words of Arne Dietrich: “It is high time that researchers became more creative about creativity” (Dietrich, 2007b).

Indeed, several attempts to turn the tide are already underway. Some researchers have, for instance, conducted functional neuroimaging investigations of creativity as it occurs in the real world during musical improvisation and story generation (Limb and Braun, 2008; Shah et al., 2011; Liu et al., 2012). Such open-ended approaches are necessarily less controlled in terms of methodology compared to close-ended approaches, but the upside is that they assess creative thinking in a more ecologically valid manner. Indeed, such tradeoffs between methodological standards and the assessment of real world versus lab creativity may be unavoidable. Another approach has been to circumscribe creativity in terms of its component processes, such as insight (Bowden and Jung-Beeman, 2007), conceptual expansion (Abraham et al., 2012b; Kröger et al., 2012; Rutter et al., 2012a,b), creative imagery (Aziz-Zadeh et al., 2012; Ellamil et al., 2012), and so on. In order to understand this approach better, we need to explore the next core question of the distinctiveness of cognitive operations involved in creativity.

## What makes creative cognition different from normative cognition?

Several neuroscientific approaches have been adopted to relate creativity to brain function. Neuroimaging, EEG and ERP investigations of creativity (for a review, see Dietrich and Kanso, 2010) seek to characterize (a) what aspect of brain function differentiates highly creative people from less creative people, and/or (b) what pattern of activity the brain exhibits when we think creatively compared to when we do not. In investigations using the former individual differences or between-subjects approach, individuals are classified as less or more creative according to their performance on a certain metric(s). Comparing brain function across high and low performing groups are held to reveal which brain-related factors have a significant impact on creative ability (e.g., Jung et al., 2010; Takeuchi et al., 2010b). The latter intra-individual or within-subjects approach holds that all human beings have the capacity to be creative and that assessing brain function during creative versus non-creative thought can reveal insights about which brain-related factors have a significant impact on creative idea generation (e.g., Green et al., 2012; Kröger et al., 2012; Rutter et al., 2012b). Other approaches include neuropsychological investigations of creativity in neurological patients (e.g., Miller et al., 1996; Seeley et al., 2008; Shamay-Tsoory et al., 2011; Abraham et al., 2012a) and psychiatric populations (e.g., Andreasen and Powers, 1975; Abraham et al., 2007) which explore the brain-related factors that are related to impaired or enhanced creativity in clinical groups relative to matched healthy controls.

What is interesting to note is that despite these efforts to understand high versus low creativity, or creative versus non-creative aspects of cognition, very little is known about how exactly creative thinking is supposed to be different from non-creative thinking. At the heart of most investigations relating creativity to brain function is the implicit idea that creative aspects of cognition are different from non-creative aspects of cognition. What is still a mystery though is the nature of this difference. Creative thinking certainly feels special. But does that necessarily mean that it is special? Such presuppositions are rarely discussed within the literature.

**Figure 1:**
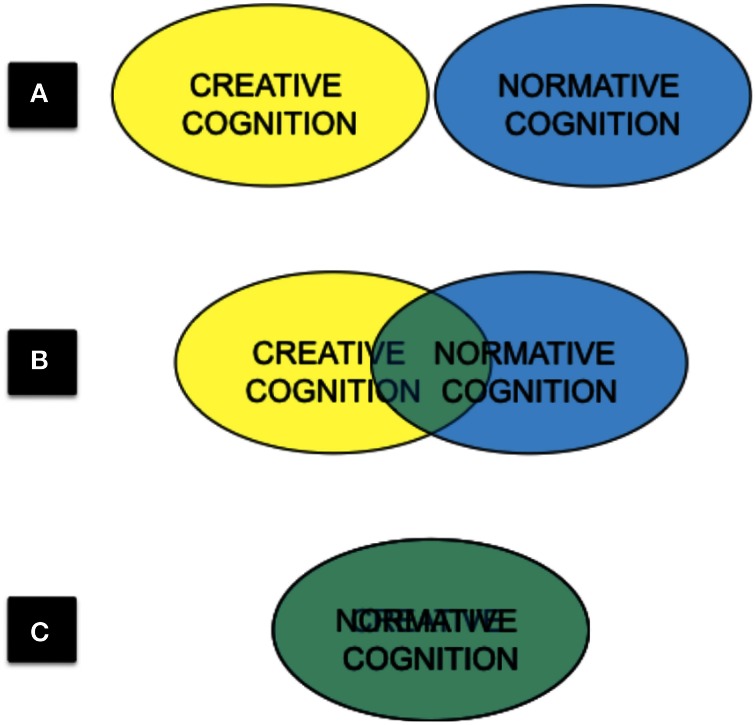
**Three conceptualizations of the relationship between creative and normative cognition: (A) mutually exclusive, (B) partially overlapping, (C) undifferentiated**.

We have much reason to presume that creativity is distinct from other aspects of cognition. Compare the creative act of writing a poem to the act of reciting the same poem from memory. It seems illogical to imagine that the cognitive processes behind the act of creating a poem are in any way congruous to those involved in memorizing it. However, if some form of qualitative distinctiveness between creative and normative aspects of cognition is assumed to exist, how are these instantiated in the brain? Is the information processing toolbox for creative cognition completely distinct from that of normative cognition (Figure 1A)? Do they partially overlap (Figure 1B)? Or are they one and the same (Figure 1C)? Again, as such issues are not debated within the field, it is impossible to adequately weigh the pros and cons of each option at this juncture.

Chief among the researchers who took a stand on such issues (e.g., Boden, 2004; Dietrich, 2004; Abraham and Windmann, 2007), were the proponents of the Geneplore model or the creative cognition approach who espoused the third view (Ward et al., 1995; Finke et al., 1996). According to this approach, the network of mental operations or the information processing toolbox is the same regardless of whether one is engaged in a creative or non-creative cognitive task. The essential difference between creative and non-creative thought processes lies in contextual (situational or task) factors that pose demands on performance. Creativity tasks necessitate generativity on the part of the subjects, as their problem contexts call for more open-ended, unstructured or non-linear information processing strategies to be adopted compared to cognitive tasks that do not necessitate creativity. For example, contrast the contextual demands involved when asking participants to report common uses for an object compared to generating novel uses. In the former case, one needs to recall different contexts in which that object was used in the past, whereas in the latter case, recalling stored information is not enough as one has to imagine new uses. The emphasis of the creative cognition approach is that one can understand the complex dynamics of creative thinking by assessing cognitive processes in contexts that are fundamentally generative in nature.

This idea fits with recent proposals concerning the interface between the fields of psychology and neuroscience which have shown that psychological states, such as thoughts and feelings, and brain states may not be instantiated in an equivalent manner to one another (Barrett, 2009; Bressler and Menon, 2010; Barrett and Satpute, 2013). This is also referred to as the “mind-brain correspondence problem” and reflects the need to understand that psychological states emerge from the interaction between more basic or primitive mental components (Barrett, 2009). Such ideas resonate with that of the creative cognition approach where several component creative cognitive operations have been operationalized (Abraham and Windmann, 2007). Conceptual expansion and insight are among the more widely investigated creative cognition components in relation to brain function (Jung-Beeman et al., 2004; Kounios et al., 2006; Aziz-Zadeh et al., 2009; Abraham et al., 2012b; Kröger et al., 2012; Rutter et al., 2012a,b), whereas research which has implications for other components like creative imagery and the constraining influence of examples is still in the nascent phase (Abraham et al., 2012a; Aziz-Zadeh et al., 2012; Ellamil et al., 2012; Fink et al., 2012).

Assumptions underlying the individual differences or between-subjects approach where the aim is to uncover the information processing or brain biases that differentiate highly creative individuals from average or low creative individuals, are also rarely questioned. Such ideas have a long history with leading theorists in the field attributing high creative ability to flat associative hierarchies in semantic knowledge (Mednick, 1962), defocused attentional processing (Mendelsohn, 1974), and cognitive disinhibition (Martindale, 1999). High creativity has been associated with a host of brain-related factors such as reduced white matter integrity in inferior frontal brain regions (Jung et al., 2010), increased grey matter in dorsolateral prefrontal and striatal areas (Takeuchi et al., 2010b), integrated white matter tracts of the corpus callosum and association cortices (Takeuchi et al., 2010a), greater right hemisphere contributions (e.g., Bowden and Jung-Beeman, 2003), increased frontal lobe activity (e.g., Carlsson et al., 2000) and heightened alpha synchronization (e.g., Fink et al., 2009).

The question that arises in such contexts is if the global brain-related differences between low and high creatives are specific to creativity (in that they have no impact on other aspects of cognition) or not. Regardless of whether this question is answered in the affirmative or the negative, the theoretical implications that derive from either stance needs to be discussed in a wider context. If such global differences have an impact on processes beyond creativity, in what other aspects of cognition would the high creatives appear to be more or less adept than the low creatives? In contrast, if such global differences specific to creativity, how is this domain-specific information processing toolbox for creativity characterized in the brain?

There is also the chicken-and-egg problem of what came first. Are the high creatives more creative because they have greater masses of grey matter in specific brain areas or does grey matter increase lead to higher creativity? Moreover, how generalizable are such findings within and across domains? Would a positive correlation between cortical connectivity and originality on a verbal creativity measure X also be expected when using another verbal creativity measure Y? How would these insights extend to performance on nonverbal measures of creativity?

A further issue of concern in this context is the choice of the control group to the high creatives. Groups are usually classified according to their performance on one or more creativity measures. However, as many creativity tasks are not comprehensive standardized (compared to IQ measures, for instance), it can be challenging to determine what the performance levels truly indicate. Does poor performance on the remote associates test, for instance, indicate below average or average creative ability? Depending on how this question is answered, the choice of which control group (low creative or average creatives) would be a better comparison group to the high creatives is a critical one that is likely to impact how one interprets the associated findings. What is also at stake is how far one can generalize such findings to the general population.

Another potentially problematic issue is the implicit idea that creativity is an inherent trait of sorts. This is because it is clear upon examining the trajectory of productivity in any individual who is considered to have reached the pinnacle of creative achievement (e.g., Picasso or Einstein), that there is considerable variability in the degree and quality of output throughout their lifetime. This suggests that creative output cannot be solely explained in terms of inherent traits but also that fluctuating or state-based aspects of creative cognition need to be considered (Harnad, 2006), even in cases of exceptional creative ability.

Some researchers assess high and low creative groups by comparing individuals who are highly proficient in ostensibly creative pursuits (e.g., art, music, dance) versus those who are not. One of the overwhelming shortcomings in faced when using this approach is to ensure homogeneity in the samples (within and between groups). What is more, the assumption that insights about creativity can be only genuinely attained by investigating people who pursue the arts compared to other professions is highly debatable for two major reasons. First, creativity is highly valued trait not just in the arts but also across a range of professions including medicine, engineering, marketing, law, advertising, research, teaching and even accounting. Second, it is fallacious to presume that anyone who pursues the fine or performing arts is, by virtue of that fact alone, guaranteed to be highly creative. Indiscriminate overgeneralizations of this nature hinder progress in the field as it propagates misrepresentations that affect our conceptualizations of creativity. This brings us to the next core question of what creativity entails.

## Do we need to distinguish between types of creativity?

To get a full sense of the current predicament faced by creativity researchers, it would be helpful to imagine the field of memory research in the absence of an established structural framework to distinguish between different types of memory. So the now well-recognized distinctions between declarative or explicit and non-declarative or implicit forms of memory would be unknown. Now imagine the task faced by any memory researcher who has to make generalizations about memory function by sifting through this rapidly growing mass of undifferentiated findings in relation to memory. Keep in mind that the findings associated with learning though conditioning (such as knowing that direct contact with fire is dangerous) would be muddled together with those of procedural skills (such as the ability to ice skate), and knowledge about facts (such as the name of your best friend in the 4th grade), as well as visuo-spatial memory (such as the way to get from your apartment to your office). It would be incredibly challenging for anybody to infer any viable ideas about memory, especially in relation to the brain, under these circumstances. Such an undertaking would allow only a vague understanding of the brain basis of memory function (e.g., the hippocampus is involved. Period.) together with the utter inability to develop a model of its information processing mechanisms. The field of memory research is greatly aided by having systematic theoretical frameworks that guide interdisciplinary empirical work. The same conditions are necessary for the field of creativity research if truly significant advances are to be made.

What is essential then is that creativity researchers develop a framework within which the myriad findings can be accurately classified. Naturally this is easier said than done and one can only start with baby steps. It is necessary to first understand what is meant by creativity before we attempt to carve this weighty concept at the joints. So what is creativity?

Creativity is defined by the presence of two components: originality (uniqueness or novelty) and effectiveness (relevance or appropriateness) (Stein, 1953; Runco and Jaeger, 2012). Originality is the key factor at the root of this concept as something that is not unusual or new in any way cannot be considered creative. Effectiveness is also vital as a response that is original but not relevant to a particular context may be considered weird or odd, but not creative.

Despite this surprisingly simple definition, the findings associated with creativity, particularly the neuroscience of creativity, can appear cluttered, inaccessible and even contradictory. One reason for this is that while this definition of creativity is readily applicable in tasks that call for some form of creative problem solving (such as in the alternate uses task), it is not as simple to apply this definition in the context of the arts. It is more difficult to obtain a consensus on both the presence and the degree of originality associated with a work of art as well as to determine to what extent the factor of effectiveness plays a role. This is ironic given that the concept of creativity is most closely bracketed with that of the arts.

Another major reason for the disorganized picture is that creativity has been assessed using different approaches by means of a wide range of tasks and even different behavioral measures within each task (Arden et al., 2010). Yet the reported findings are rarely circumscribed in terms of their specific implications in relation to select aspects of creativity (e.g., fluency in creative idea generation) and are instead, indiscriminately presented as concerning creativity as a whole. Other problems include the questionable equivalency when adapting established creativity tasks, such as the remote associates test, for use in non-English speaking contexts (for a lengthy discussion on this issue, see Abraham et al., 2012a).

There are a plethora of research topics related to creativity but very little debate or discussion to bring the different areas together. These include musical creativity, visual creativity, synesthesia and creativity, divergent thinking, insight and convergent thinking, scientific creativity, artistic creativity, verbal creativity, creative problem solving, creativity and analogical reasoning, and creativity and metaphors. Further complications result because it is unclear how creativity in one domain relates to creativity in other domains. For instance, it would be useful to know if (and how) insights on “visual creativity” extend to “artistic creativity” but not “verbal creativity”. This is, of course, difficult to do because we lack a unifying framework within which widely disparate facets of creativity can be brought together.

**Figure 2:**
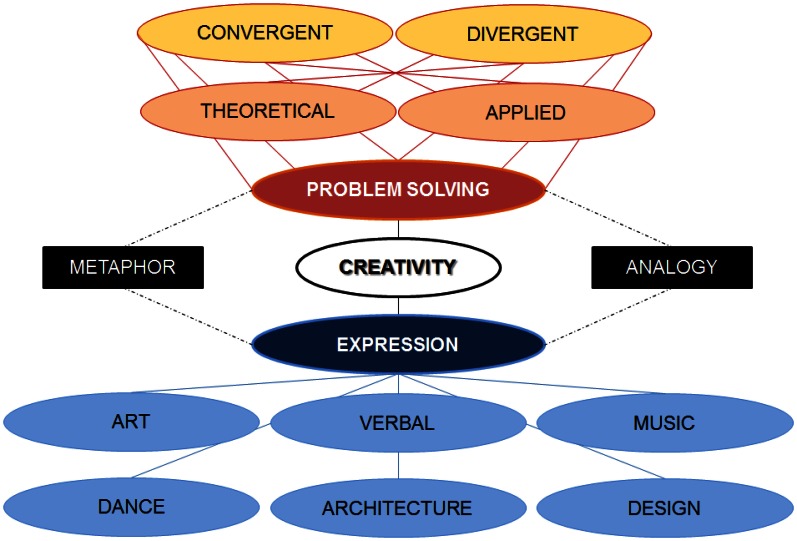
**A preliminary framework to distinguish between branches of creativity: problem solving domain and the expression domain**. There are subdomains (within oval forms) within each domain and strategies (within rectangular forms) common to both domains.

A first step that can be taken to aid the development of such a framework would be to assess neuroscientific studies of creative thinking in terms of determining the implications of their results for a particular branch of creative thinking. One possibility would be to assign the studies as falling under one of two basic branches or domains of creativity: problem solving and expression (Figure 2). Problem solving abilities are customarily assessed by performance on one or more creativity measures that assess analytical thinking (Grabner et al., 2007; Jung et al., 2010), whereas expression abilities are mainly determined by the degree of proficiency in a particular domain (e.g., art, dance) with high expressive ability being defined as above average (Fink et al., 2009; Gibson et al., 2009) or exceptional (Hou et al., 2000; Snyder, 2009). The goal in the case of creative problem solving is to develop novel solutions to problems whereas the goal in the case of creative expression is to express oneself in a unique manner. The underlying commonality between creative problem solving and creative expression domains would be that both involve problem finding, problem creation or problem identification (Getzels, 1975; Csikszentmihalyi and Getzels, 1988), which is central to creative thinking. However, they differ in terms of contextual factors which can be classified as predominantly problem solving based or, alternatively, predominantly expression based. The word “predominantly” is employed here to make apparent that the problem solving and expression domains are not mutually exclusive.

The problem solving and expression domains can be further classified into subdomains. For instance, separate expression subdomains can be envisioned for artistic, musical, kinesthetic and verbal skills. Each of these can be further subdivided into meaningful categories and this would be best done in consultation with researchers with the necessary expertise in research on these expression and proficiency aspects of creative thinking.

The problem solving domains could be subdivided in a similar manner. The classic division of convergent versus divergent thinking may be applicable here. In creative convergent thinking, the means-state and end-state of the problem is known but the path to get from the means-state to the end-state is not linear and necessitates conceptual restructuring. Successful restructuring is commonly associated with the phenomenon of insight. In creative divergent thinking, in contrast, the end-state is unknown and there is more than one potential solution to the problem. So the path to the end-state has to be charted and the end-state itself has to be conceived. There is no reason to assume that the process of insight is limited to convergent contexts, as it would be expected to accompany any situation where a novel connection is forged between previously unrelated or weakly related concepts. It may be viable to also distinguish between theoretical versus applied subdomains in the problem solving domain with domains such as mathematical and physics falling under the theoretical domain, whereas medicine and engineering fall under the applied domain.

It is possible that cognitive strategies to generate ideas may be commonly applied across creative problem solving and expression domains but that the contextual factors differ according to the domain in question. For instance, recent meta-analyses were conducted to dissociate metaphor and analogy components of creative thinking (Vartanian, 2012). Such strategies are commonly used during creative problem solving, but it is also plausible that this metaphor/analogy strategy division can be readily applied in domains of creative expression, such as verbal creativity and artistic creativity. Using such strategies as a common metric, it would be possible to chart parallels and distinctions in neurocognition across the creative problem solving and creative expression domains.

This proposal to dissociate the problem solving and expression domains is based on preliminary ideas. No doubt there will be a lot of stumbling as attempts are made to develop these baby steps. Yet it is essential that we nonetheless attempt to formulate a workable framework in order to develop a comprehensive understanding of the neurocognitive mechanisms underlying creativity. Differentiating problem solving from expression domains in creativity would mean that insights obtained by, for instance, investigating the brain connectivity in absolute pitch musicians as a mark of musical creativity (Loui et al., 2011) or frontotemporal dementia patients who develop unprecedented artistic skills post-stroke (Seeley et al., 2008), cannot be blindly generalized to findings associated with studying brain activations that result while performing the alternate uses task (Chrysikou and Thompson-Schill, 2011). Such a view in no way advocates that selective findings should be ignored. Quite the contrary. Any claim that a study's findings are relevant to any domain of creativity needs to be assessed in terms of how it fits into the larger framework of creativity.

As such a framework is currently lacking, the onus is on us researchers to carry out our investigations with these larger objectives in mind. So, for instance, when drawing allusions between the expression and problem solving subdomains in creativity, it would be useful to indicate how expression-based findings, such as enhanced drawing skills following transcranial magnetic stimulation (TMS) (Snyder et al., 2003) can be explicitly linked to problem-solving-based findings, such as greater originality following cognitive stimulation (Fink et al., 2010). The division between these creative problem solving and creative expression domains may certainly be more fluid than we imagine and the associations between them beg further exploration. Research efforts that come close to bringing both these domains together, such as evaluating the brain response during jazz improvisation or freestyle rap in proficient musicians (Limb and Braun, 2008; Liu et al., 2012), will aid us in reaching this aim.

Developing a classification system by which to approach various aspects of creativity would help us deal with further critical issues such as formalizing the differences and similarities between the diverse types of creativity as well as operationalizing the many creativity tasks in terms of what proportion of cognitive operations are shared between them. These are necessary paths to explore if the central objective of the neuroscience of creativity is to glean the underlying brain and information processing mechanisms of this most extraordinary of human abilities.

## Conclusions

This treatise explores many of the current challenges faced by neuroscientists in the field of creativity. Although we face considerable impediments, progress will necessarily certainly be made in the field as long we confront the issues head on. There is a dire need for more research groups all over the world to become involved in the investigation of creativity. The necessity for collaborative efforts has never been stronger. The ideal way to kick start such a venture would be to organize a series of workshops which features exchanges between experts from the field of creativity and those from relevant fields of psychology and cognitive neuroscience (semantic cognition, imagery, etc.) to exchange ideas and work together to develop a theoretical framework for the neuroscience of creativity. It certainly is time for us to be more creative about creativity. What better way than to move the focus from individuals developing ideas in isolated cocoons to developing our ideas together in a common space, where stimulating interchanges are guaranteed and where our hunches can develop and be nurtured by other ideas (Johnson, 2011).

### Conflict of interest statement

The author declares that the research was conducted in the absence of any commercial or financial relationships that could be construed as a potential conflict of interest.

## Footnotes

^1^Online sources for information regarding fMRI design related issues include: (1) http://mindhive.mit.edu/book/export/html/66 (2) http://imaging.mrc-cbu.cam.ac.uk/imaging/DesignEfficiency (3) http://afni.nimh.nih.gov/pub/dist/HOWTO//howto/ht03_stim/html/stim_background.shtml.
